# Erythematous pustules and plaques following a dental procedure

**DOI:** 10.1016/j.jdcr.2024.07.038

**Published:** 2024-08-24

**Authors:** Geetha Gowda, Seanna Yang, Giuseppe Tripodi, Howard Ragland, Andrea Murina

**Affiliations:** Department of Dermatology, Tulane University School of Medicine, New Orleans, Louisiana

**Keywords:** actinomycosis, anaerobic bacteria, dental infection, histopathology, mycetoma, pathology, pustules, skin infection

## Case report

**Fig 1:**
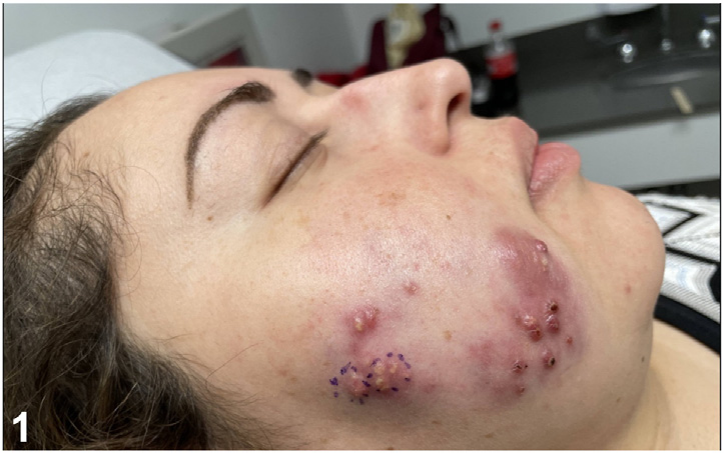

**Fig 2:**
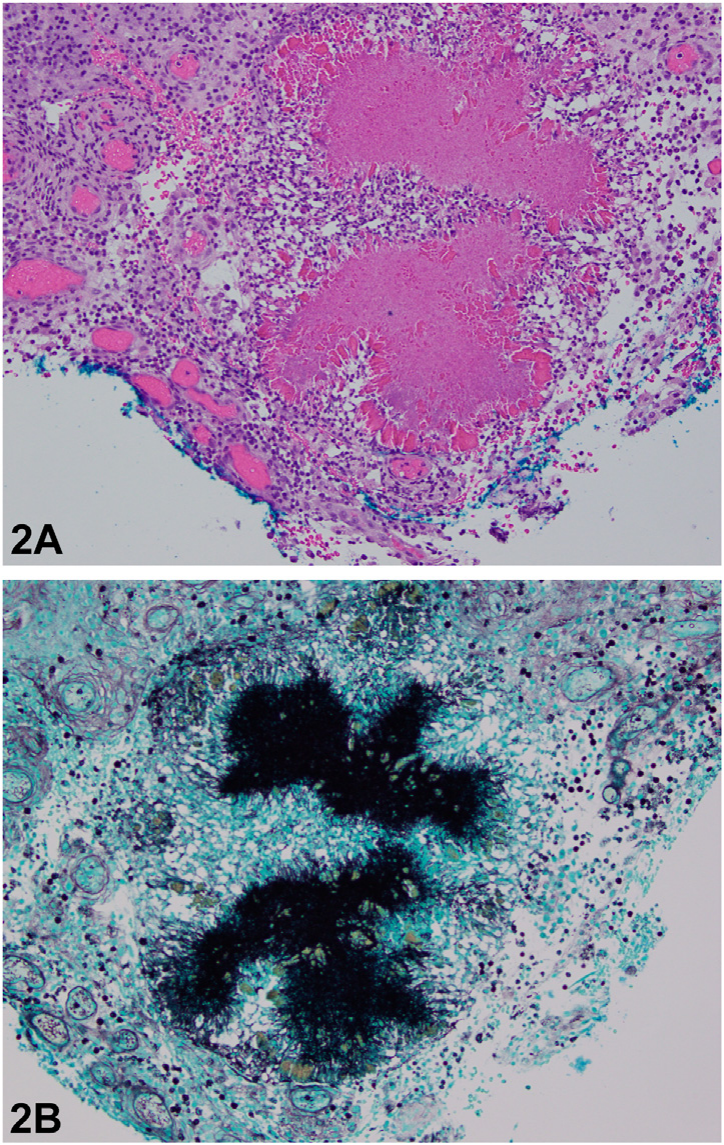

A 27-year-old multigravida female at 22 weeks gestation presented to the clinic for evaluation of an 8-month history of painful, erythematous pustules and indurated plaques to the right cheek (Fig 1). Four months before the onset of symptoms, she underwent a dental filling procedure complicated by postoperative infection, which self-resolved. The patient managed her symptoms at home by occasionally draining the areas with an alcohol-cleansed needle, which resulted in yellow discharge. No other topical or systemic therapies were tried. Histopathology and special stains were performed (Fig 2, *A* and *B*).

**Question 1: Which of the following is the most likely diagnosis?**

A. Actinomycosis
B. Nocardiosis
C. Mucormycosis
D. Blastomycosis
E. Aspergillosis

**Answers:**

A. Actinomycosis – Correct. *Actinomyces* is a slow-growing bacteria that requires an anaerobic environment, which can be missed on routine aerobic culture. Histologically, *Actinomyces* is a nonacid-fast gram-stain-positive filamentous rod. Cervicofacial actinomycosis presents with nonspecific symptoms that can mimic granulomatous disorders and cancers. Notable risk factors for the development of cervicofacial actinomycosis involve penetration of the oral mucosa, generally caused by oral surgery, radiation therapy, and dental caries.^1^
B. Nocardiosis – Incorrect. Nocardiosis can present with pulmonary, cutaneous, and central nervous system manifestations, especially in immunocompromised patients. Histologically, it stains as gram-positive filamentous rod, similar to Actinomyces. It differs from *Actinomyces* by its aerobic pattern of growth.^1^
C. Mucormycosis – Incorrect. Mucormycosis can invade blood vessels, leading to thrombosis and tissue necrosis. Cutaneous mucormycosis can appear erythematous to violaceous plaques that can progress to necrosis or an eschar. Histologically, mucormycosis would present as pauci-septate hyphae with wide angle branching.^2^
D. Blastomycosis – Incorrect. Disseminated blastomycosis can commonly have cutaneous involvement presenting as verrucous or ulcerated pustules or plaques. Primary cutaneous blastomycosis is rare and presents as papules or pustules with regional lymphadenopathy.^3^ Histologically, blastomycosis would present with broad-based budding yeasts.^2^
E. Aspergillosis – Incorrect. Aspergillosis typically presents with pulmonary involvement and can have secondary cutaneous manifestations presenting as ulcerated papules and plaques. Cutaneous involvement typically presents in immunocompromised patients.^3^ Histologically, aspergillosis presents with septate hyphae with acute angle branching.^2^

**Question 2: What is the most appropriate treatment in this scenario?**

A. Hydrocortisone 2.5% ointment
B. Prednisone
C. Amoxicillin-clavulanate
D. Trimethoprim-sulfamethoxazole (TMP-SMX)
E. Ursodeoxycholic acid

**Answers:**

A. Hydrocortisone 2.5% ointment – Incorrect. This would be used in an inflammatory condition of pregnancy, such as an atopic eruption.^4^
B. Prednisone – Incorrect. Systemic corticosteroids can be used to treat pemphigoid gestationis, a rare bullous dermatosis related to pregnancy.^4^
C. Amoxicillin-clavulanate – Correct. Most *Actinomyces* infections are sensitive to beta-lactams, and the treatment of choice is a prolonged course of amoxicillin-clavulanate. Intravenous preparations can be used in severe cases. Alternatives to amoxicillin-clavulanate include clindamycin, macrolides, and doxycycline. Additionally, if dental caries or abscess are the cause of the infection, dental treatment with extraction of the tooth is necessary. Surgical management may be required for draining abscesses or debridement of necrotic bone tissue if the infection progresses to osteomyelitis.^1^ The patient presented in this case was treated with a 6-month course of amoxicillin-clavulanate and underwent extraction of the right first upper molar. The patient demonstrated interval improvement in symptoms at follow-up visits.
D. Trimethoprim-sulfamethoxazole (TMP-SMX) – Incorrect. TMP-SMX can be used to treat bacterial infections such as nocardiosis.^5^ It is typically avoided during the first trimester of pregnancy.
E. Ursodeoxycholic acid – Incorrect. Ursodeoxycholic acid is a bile acid that can be used to treat intrahepatic cholestasis of pregnancy.^4^

**Question 3: What is a histological finding in Actinomycosis?**

A. Mycetoma
B. Aspergilloma
C. Endospore-filled spherules
D. Encapsulated yeast cells
E. Pseudohyphae

**Answers:**

A. Mycetoma – Correct. The typical histological appearance of an actinomycete infection is a central neutrophilic abscess with sulfur granules surrounded by granulation tissue with radiating filaments.^5^
B. Aspergilloma – Incorrect. Aspergillomas are seen after infection with *Aspergillus fumigatus.* The histological presentation appears as septate hyphae with acute-angle branching.^2^
C. Endospore-filled spherules – Incorrect. Endospore-filled spherules are a classic histologic finding following infection with *Coccidioides.* These spherules can rupture releasing yeast throughout the body.^2^
D. Encapsulated yeast cells – Incorrect. *Cryptococcus neoformans* presents as encapsulated yeast cells on India ink stain. This is an invasive fungus that can cause meningitis in immunocompromised individuals.^2^
E. Pseudohyphae – Incorrect. *Candida albicans* can present as pseudohyphae on Gomori methenamine silver stain. The fungus is dimorphic, and it can present as a yeast or hyphal form.^2^

## Conflicts of interest

Dr Murina is a speaker for Abbvie, Amgen, Bristol-Meyers-Squibb, and Janssen. She has served as a consultant for Bristol-Meyers-Squibb, Janssen, Novartis, Ortho-Dermatologics, Takeda, and UCB. She is on the Editorial Board of the JAADCR. Drs Gowda, Yang, Tripodi, and Ragland have no conflicts of interest to declare.
